# Supplementary material for: Are carbon emissions trading and green financial instruments synergistic? -Comprehensive quantitative research based on content analysis
Source: PLoS One. 2024 Mar 7;19(3):e0298601. doi: 10.1371/journal.pone.0298601 (PMC10919601; doi:10.1371/journal.pone.0298601)
Supplement: S1 Data — (ZIP) [file pone.0298601.s001.zip › Data/地级市Z_F工作报告.zip!/║■─╧/╔█╤⌠╩╨/└┤╫╘╣1⁄2╓┌║┼í╛┬φ┐╦╩2╛▌═°í┐_2018.docx]

各位代表：

现在，我代表市人民政府，向大会报告政府工作，请予审议，并请政协各位委员提出意见。

一、2017年工作回顾

2017年，面对严峻复杂的经济下行压力和历史罕见的洪涝灾害，在省委、省政府和市委的坚强领导下，我们坚持稳中求进工作总基调，抓重点、补短板、强弱项，积极应对，攻坚克难，较好完成了市十六届人大一次会议确定的年度目标，实现了本届政府工作的良好开局。

（一）狠抓政策落地，经济运行稳中有进。认真贯彻落实中央和省委、市委关于稳增长和供给侧结构性改革工作部署，落实降低实体企业成本21条、出台迎老乡回家乡创业发展15条、房地产去库存19条和物流业降本增效若干政策措施，促进经济稳步发展。市财政拿出3亿元作为企业续贷“过桥”资金，中小企业融资担保资金由1.1亿元扩充到3亿元，帮助实体企业渡过难关。“三去一降一补”成效初显。降低企业用能、物流成本5.1亿元，减轻企业税费负担15.1亿元。新入规“四上”企业726家、排名全省第2位，其中规上工业企业194家、排名全省第3位，其它专业入规数量排全省第2位，5000万元以上投资项目98个、排全省第4位。推动280家停产减产企业复产达产。棚户区改造开工46309户，货币化安置比例提高到60%，争取棚改贷款102亿元，是上年的2.6倍。商品房去化周期由24.9个月缩短到15.2个月。争取中央和省预算内资金30亿元，完成政府性债务置换100.3亿元。城投全年完成投融资240.2亿元，同比增加111.9亿元，为一批重大项目实施提供了有力支撑。经济质量有所提升。产业结构由上年的21.5：35.2：43.3优化为19.7：35.4：44.9，一产比重下降1.8个百分点，历史性首次降到20%以下，二产比重提高0.2个百分点，三产比重提高1.6个百分点。战略性新兴产业总产值同比增长12.5%，新增高新技术企业33家，实现高新技术产业增加值300亿元，同比增长18%。专利授权1627件，有效发明专利拥有量340件，“湘中”商标被国家工商总局认定为中国驰名商标。全市有效商标注册量突破2万件，居全省第2位。新增财政收入过10亿元县1个。全市地方财政收入税占比首次上升到50.17%，上升5.62个百分点。经济整体稳中向好。面对高开低走、低迷下沉的重重压力，我们对症施策、密集调度、精准发力，有效遏制严重下滑，成功实现逐步拉升。全年实现地区生产总值1691.5亿元，增长8.1%；固定资产投资1840.1亿元，增长13.1%；财政总收入153.4亿元，增长8.7%；规模工业增加值535.1亿元，增长7.3%；社会消费品零售总额926.2亿元，增长11.0%；进出口总额114.7亿元，增长33.1%；实际利用外资2.66亿美元、增长18.1%，实际利用内资330.8亿元、增长17.5%；城乡居民人均可支配收入16353元，增长10.8；城镇居民人均可支配收入25029元、增长8.8%，农民人均可支配收入10756元、增长10.6%。11项主要经济指标增速，8项高于全省，其中5项排全省前4位，2项与全省持平，1项（进出口总额）低于全省。

（二）狠抓实体产业，转型升级初见成效。全力推进“产业兴邵”，出台产业发展实施意见和考核办法，组建14个项目推进小组，推动生产要素向主导产品、优势产业集聚，实体产业投资增速由负转正，全年增长1.8%。工业转型升级。全市第一批重点产业项目完成投资296.4亿元。全市工业园区完成固定资产投资295亿元，增长20.5%；技工贸总收入2157.8亿元，增长14.2%。湘商产业园竣工厂房使用率超过91%，新增签约企业170家，新投产200家，新增用工2万人，实现产值140亿元、税收6.5亿元，在全省“135”工程三年综合评价考核中名列第一。主导产业由过去的轻化型、能耗型开始向智能型、绿色型转变。邵东锐科机器人在中国青年创新创业板挂牌上线，智能家电小米电饭煲、邵东智能制造、3D打印等一批新兴产业项目建成投产或单项投产，彩虹集团邵阳特种玻璃、桑德集团、富电集团、湘电集团等一批重大产业项目签约落户，皮具箱包、印刷、小五金、打火机、品牌服饰、发制品等一批传统特色产业联建园区、开始成建制升级。农业提质增效。粮食播种面积和产量保持稳定，其中优质稻种植面积116.5万亩，比前三年平均数增加37%。生猪、肉牛、奶牛饲养量全省第一。良种油茶、柑橘、中药材等特色经作快速发展。“三品一标”认证产品206个，农产品加工企业发展到5623家，加工销售收入1043亿元。邵阳县国家油茶产业示范园建成运行，“邵阳油茶”入选第一批中国特色农产品优势区。隆回军杰蔬菜示范产业园等6个园区进入首批全国农村创业创新园区目录。服务业活力凸显。友阿国际广场建成营业，世界500强沃尔玛入驻邵阳。邵东大市场、湘西南农产品物流中心、步步高新天地等44个现代商贸物流项目加快推进。成功举办中国·邵阳油茶产业精准扶贫暨油茶互联网博览会、中国·邵东首届国际五金机电博览会。邵东、城步、洞口、新邵、邵阳县获批全国电子商务进农村综合示范县。积极发展全域旅游，魏源故居等8个旅游景区获评国家AAA级旅游景区，接待游客数量、实现旅游收入较快增长。金融服务业发展迅速，全年新增各类金融机构35家，存贷比提高2.9个百分点。

（三）狠抓项目建设，有效投资不断增长。突破征地、拆迁、融资和要素供给等瓶颈制约，实施重点建设项目287个，完成投资561亿元，超额完成年度计划2.1个百分点，150个项目实现全部投产或单项投产。48个省重点建设项目完成投资428.8亿元，为年度计划的112.8%。开展“奋战五个月，喜迎十九大，建设新邵阳”重点项目攻坚行动，一批重点项目相继建成或开工。武冈机场2017年6月28日通航，陆续开通北京、郑州、长沙、海口、昆明、杭州、重庆等7个城市的航线，邵阳由“高速时代”进入“高铁时代”，进而迈入“航空时代”。武靖高速邵阳段建成通车，洞兴高速全线贯通，我市又新增一条出省大通道。怀邵衡铁路邵阳段加速推进，今年可望竣工通车。投资40亿元的现代化邵阳站综合交通枢纽项目开工，坪上综合客运枢纽主体工程完工。犬木塘灌区（神滩替代）工程、城市第二水源工程项目前期继续推进。建设干线公路424公里，建成通车200公里；完成农村公路提质改造1531公里。建成光伏扶贫项目281个。“气化邵阳”一期工程市区—邵东、市区—邵阳县全线贯通，即将投产供气。

（四）狠抓基础设施，城乡建设持续推进。投资75亿元，推进市城区基础设施建设三年行动，全市城镇化率提高到45.9%。雪峰大桥、桂花大桥竣工通车，邵西大道南段、湖口井路、新城大道西段建成通车，学院路二期、邵水东路南段、邵水西路茅坪段稳步推进，市三环线虎形山路、东城快线、魏源西路、桃花桥、资江北路、蔡锷大道（邵九公路）等一批城市路网工程相继开工，紫霞公园、体育公园、北塔公园等一批城市公园启动建设。市体育中心及周边路网、市污泥集中处置中心、市燃气安全指挥中心等重点民生工程建设全面提速。邵阳第一座220千伏智能GIS变电站元宝变顺利投产；371个自供区村农网改造全部完成，实现同网同价。中心城区数字化城管投入使用，主次干道“白改黑”全覆盖。一批城市骨架工程、配套工程相继建成或启动，中心城区基础设施进一步完善，管理水平进一步提升，成功创建国家卫生城市。积极推进全国文明城市创建，在全省参与城市文明程度指数测评的五个市州中排名第一。下花桥镇入选全国特色小镇，堡面前乡大羊村入选全国改善农村人居环境示范村，黄桥镇、滩头镇、坪上镇、五峰铺镇被评为全省美丽乡镇示范镇，山门镇、长铺子乡田心村、李熙桥镇陈家村、山界乡老屋村、双牌镇浪石村被评为省级历史文化名镇名村，建成省级美丽乡村28个，新建美好社区212个。

（五）狠抓改革开放，市场主体活力增强。推进“放管服”改革，全面清理行政审批事项，完成权力清单、责任清单、负面清单编制，权力事项精简60%，非行政许可审批事项全部取消。完成经开区托管乡（镇）村改革，向邵阳经开区一次性下放经济管理权限232项。推进南山国家公园体制改革试点，湖南南山国家公园管理局正式挂牌，总体规划基本完成，公园自然资源确权登记发证开始试点。深化商事制度改革，“多证合一”全面施行，新登记市场主体22015户，增长17.53%。城管综合执法体制改革全面完成。农村土地承包经营权确权登记颁证工作稳步推进。创新非税收入管理，我市成为全国首个通过电子凭证库实现非税收入缴库城市。市不动产数字管理技术创新项目获湖南省首届“管理创新奖”。市公共资源交易中心取消业主评委等多项创新实践在全省推广。成功举办第三届“邵商大会”，组团参加“港洽周”、深圳招商推介会、“湘商大会”，积极对接“三个500强”，各产业小组主动对接500强实体企业26家，招商引资、外经外贸和对外合作成效明显。湖南出入境检验检疫局邵阳办事处机构获批，进出口通关服务中心开工建设。保税仓建设运营改革试点全面启动，邵阳经开区公共保税仓开工建设。聘请李国杰院士等12名专家为邵阳市科技经济顾问。

（六）狠抓环保整治，生态环境明显改善。积极配合中央环保督察，国省交办的222件信访件办结221件，有效回应了人民群众对生态环境改善的关切。共关闭企业360家，责令整改235家，淘汰落后产能项目16个，对有污染的10家企业转产退出，对湘中制药等6家发展前景好的企业“退城入园”。全面淘汰市区所有10蒸吨以下燃煤锅炉，全面禁止市城区燃放烟花爆竹。新增新能源公交车472辆，累计淘汰黄标车11825辆；为淘汰黄标车，市财政一次性支出5.8亿元。推动机动车尾气污染物检测。完成枫江溪、邵水（市区段）、红旗河3条黑臭水体治理。完成梅子井、珑瑚排污口污水截留处理，结束了中心城区水源保护地污水直排历史。完成龙须塘区域环境综合整治3个重金属点源治理。粘土砖厂全部关闭，禁养区内养殖场全部退出。环保整改中，共立案处罚48家，罚款237.7万元，立案侦查19家，行政拘留9人，刑事拘留14人，对履职不到位的公职人员约谈92人、问责112人。强化农村环境综合整治，启动重点镇污水处理设施建设18个，新建无害化垃圾焚烧炉206座、垃圾中转站65座。继续实施“四边五年”绿色行动，完成造林37.6万亩，全市森林覆盖率达到60.49%，城步、新邵获评“全国绿化模范县”。全面落实四级“河长制”，有效整治污水直排、垃圾倾倒、无序采砂、网箱养鱼、挤占堤岸等顽症痼疾，水质水岸明显好转，“一河一档”、“一河一策”全面启动，在全省率先推进“僵尸船”清理拆解工作。

（七）狠抓脱贫攻坚，民生福祉保障有力。强力推进脱贫攻坚，投入各类扶贫资金64亿元，可望实现21万人脱贫、441个贫困村“摘帽”。建成金融扶贫服务站1074个，新增扶贫小额贷款10.3亿元。建设易地扶贫搬迁安置住房9914套、搬迁37757人，完成危房改造14126户。完成人安饮水工程1074处，解决22.57万贫困人口安全饮水。发放农机补贴3713万元、受益农户25259户。完成农村公路“窄改宽”1139公里。建成和在建、改建村卫生室2943个，实现全市所有贫困村标准化卫生室全覆盖。新建和在建、改建村级综合服务平台2559个、乡镇综合服务平台135个，实现全市农村综合服务平台全覆盖。大力发展各级各类教育，合格学校完成率99%。湘中幼儿师范高等专科学校建成并招生入学，市一中搬迁项目开工建设。市中心医院东院试运营。市体育中心项目主体工程完工，市博物馆、文化馆、图书馆、美术馆、规划馆相继建成开馆。市第四届少数民族传统体育运动会在隆回成功举办。新增城镇就业5.1万人，失业率控制在4.5%以内。各项民生支出425.9亿元，增长31.4%，占财政总支出81.4%，均创历史新高。农村最低生活保障标准提高到3026元/年，连续12年调高企业退休人员基本养老金，连续4年提高城乡居民养老保险基础养老金。发放住房租赁补贴3.3万户、3366万元。新增发放住房公积金个人贷款24亿元，市场占有率达46%。

（八）狠抓综合治理，社会大局和谐稳定。2017年6月下旬至7月初，我市遭遇历史罕见的长时间持续强降雨，过程强度历史第二，局部降雨量、降雨强度超历史极值，造成全流域大面积受灾。市委市政府沉着应对、科学调度，驻邵官兵勇挑重担、冲锋在前，全市上下众志成城、日夜奋战，取得了抗洪救灾的决定性胜利。严格落实安全生产责任制，深入开展重点行业、重点领域安全生产专项整治和打非治违专项行动，全年没有发生重特大事故，各类事故起数、死亡人数分别下降61.39%、53.89%，安全生产工作再获全省先进。食品药品安全监管持续发力，安全形势稳中趋好。完成省际市际长途客运实名制工作。推进“公安消防大建设三年行动计划”，加强“一村一辅警”建设，完善社会巡逻防控，屯警街面、动中备勤，全市没有发生重大群体性事件和重特大恶性案件，社会治安持续平稳，综治民调排名全省第八。“七五”普法顺利实施，社区矫正和安置帮教工作得到加强。落实信访维稳工作责任，一批矛盾纠纷和信访积案得到化解，特别防护期维稳安保工作再创佳绩。加大文明单位创建力度，新增“全国文明单位”5个。

一年来，我们扎实推进“两学一做”学习教育常态化、制度化，认真执行市人大及其常委会的决议决定，自觉接受市人大的法律监督、工作监督，主动接受市政协的民主监督，158件人大代表议案、建议和286件政协提案全部办结，见面率100%、满意率99.4%。积极推进法制政府建设，严格执行重大行政决策程序规定，科学决策、民主决策进一步加强。村级换届圆满完成。推进廉洁政府和高效政府建设。严禁严查公职人员涉矿、涉砂、涉渔、涉小水电、涉烟花爆竹、涉危险化学品六类经济活动。深入开展“雁过拔毛”、“禁赌限牌”和“四治四提”等专项整治，重点整治群众身边的腐败问题和干部不作为、乱作为、慢作为问题，群众对政府的满意度进一步提高。推进审计监督全覆盖，查出各类违规资金44亿元。开展政府投资审计，为财政节约建设资金3亿元以上。

档案、民族、宗教、海事、人防、国安、双拥、国防动员、国防教育、供销、气象、邮政、通讯、工会、共青团、妇女儿童、科协、残联、文联、工商联、台湾事务、外侨港澳事务、红十字会、驻外办事机构、高等院校、科研院所等工作取得新的成绩。

各位代表！一年的成绩来之不易，是市委统揽全局、正确领导的结果，是市人大、市政协、邵阳军分区及驻邵部队、武警官兵和社会各界戮力同心、鼎力支持的结果，是全市人民团结合作、奋力打拼的结果。在此，我代表市人民政府向全市人民和各级干部，向所有关心、支持邵阳发展的同志们、朋友们，表示衷心的感谢，并致以崇高的敬意！

在总结成绩的同时，我们也清醒地看到不足：实体经济发展困难，个别经济指标没有达到预期，稳增长难度越来越大；传统产业转型升级缓慢，新兴产业战略支撑能力不强，经济发展新动能不足；项目建设要素制约突出，推进力度不够，重大项目较少，投资持续增长乏力；脱贫攻坚任务艰巨，财政收支矛盾突出，民生保障压力较大；创卫成果时有反弹，空气指数全省排名靠后，污染治理、生态修复任重道远；一些部门和少数干部服务意识不强，攻坚克难动力不足、能力不够，缺乏责任担当。对此，我们将坚持问题导向，采取有力措施，不断加以解决。

二、2018年工作安排

2018年政府工作的总体要求是：以习近平新时代中国特色社会主义思想为指导，全面贯彻落实党的十九大精神，牢牢把握社会主要矛盾变化新趋势和稳中求进工作总基调，坚持新发展理念，坚持以人民为中心的发展思想，按照高质量发展要求，以供给侧结构性改革为主线，大力实施“创新引领，开放崛起”战略，加快推进“二中心一枢纽”建设，突出产业兴邵，努力改善民生，坚决打好防范化解重大风险、精准脱贫和污染防治三大攻坚战，促进经济社会持续健康发展。

经济社会发展预期目标是：GDP增长8%，规模工业增加值增长8%，固定资产投资增长14%，财政总收入增长8%以上，社会消费品零售总额增长11%，实际利用外资增长12%，实际利用内资增长15%，进出口总额增长15%，城镇居民人均可支配收入增长8.5%，农民人均可支配收入增长10.5%，人口自然增长率控制在9‰以内，居民消费价格涨幅保持在3%左右，万元GDP能耗下降3.5%。

围绕以上工作要求和目标任务，重点抓好六个方面的工作：

（一）提质增效，全力振兴实体经济

中国特色社会主义进入新时代，我国经济发展也进入了新时代，基本特征是经济由高速增长阶段转向高质量发展阶段。高质量发展就是能够很好满足人民日益增长的美好生活需要的发展，是体现新发展理念的发展，是创新成为第一动力、协调成为内生特点、绿色成为普遍形态、开放成为必由之路、共享成为根本目标的发展。要认真学习、深刻领会、全面落实习近平新时代中国特色社会主义经济思想，矢志不移推动高质量发展，推动质量变革、效率变革、动力变革，加快建设实体经济、科技创新、现代金融、人力资源协同发展的产业体系。经济发展的根本在实体、在产业。要坚持以园区为载体、以产业链为抓手、以优化环境为基础、以项目建设为关键、以引才聚才为支撑，着力培育、发展、壮大、做优实体产业。紧紧围绕产业发展、对外开放、脱贫攻坚、环境治理、投资环境改善等短板、弱项，集中力量攻坚克难，把重点落实，把短板拉长，把弱项变强。

全力以赴抓产业。要立足邵阳“一带一部”新定位，对接全省“四大体系”、“五大基地”建设，瞄准20个优势产业链和“五个100”产业布局，重点推进市委确定的“一走廊六基地”产业建设。“一走廊”：就是建设沪昆高速百里工业走廊。以邵阳经开区和湘商产业园为平台，在培育壮大现有企业的基础上，积极引进一批重点产业项目及配套产业链，打造湘中湘西南规模最大、覆盖人口最多的百里工业走廊。“六基地”：一是建设智能制造基地。顺应科技革命和产业变革新趋势，抓住智能制造这个核心，提质升级传统制造业，加快培育壮大新兴产业，大力促进互联网、大数据、人工智能同实体经济的深度融合。围绕省里主推的20个工业新兴优势产业链，力争在工程机械、显示功能材料、先进储能材料、新能源装备、3D打印及机器人等5个重点领域率先突破，推动我市制造业加速向网络化、智能化、绿色化方向延伸拓展，努力在智能制造方向“杀出一条血路来”。以信息化、智能化提升传统工业，用人工智能（机器人、机械手）改造打火机、小五金、皮具箱包、制鞋、服饰等行业的制造工艺，着力提升产品质量和劳动生产率。以标准化、品牌化发展特色产业，推进发制品产业园建成投产，并由档发向品牌提升。以集成化、链条化壮大优势产业，重点围绕三一智能渣土车和搅拌车、亚洲富士电梯、恒天九五邵阳纺织机械、智能家居、盖板玻璃、汽车零配件等装备制造龙头企业，吸纳其产业链上下游配套企业来邵集聚发展，打造先进装备制造业基地。排除一切阻力，克服一切困难，砸锅卖铁也要尽快把特种玻璃、拓浦精工智能制造、富电新能源、桑德环保、湘电制造等一批已落户的战略性新兴产业做起来，全力扶持其中一、二家优势企业在2020年成功冲刺百亿企业。二是建设优质农产品基地。实施“一县一主导产业和产品”，重点建设市区近郊蔬菜和苗木花卉、邵东中药材、新邵富硒农产品、邵阳县油茶、隆回金银花和龙牙百合、洞口蜜橘和茶叶、武冈卤制品、新宁脐橙、绥宁南竹、城步奶业和绿色有机蔬菜等优质农产品供应基地，推进农产品精深加工，努力实现农产品规模化、品牌化、效益化。三是建设文化产业基地。大力传承和发展传统戏曲、传统工艺、民俗风情等非物质文化遗产，加快魏源文化、花瑶文化、君子文化等地方特色文化品牌建设，积极引进一批国内外知名文化企业和重大文化产业项目，培育壮大一批龙头文化企业，努力打造湘中湘西南文化产业园和游乐中心。四是建设全域旅游基地。现在邵阳高铁通了，机场有了，新桂高速、武靖高速开通了，怀邵衡动车也将开通，这就有了很好的交通基础。我们要利用世界自然遗产崀山、南山国家公园、六月六山歌节、四八姑娘节、花瑶“讨僚皈”节等旅游品牌，积极对接张家界、桂林等国际旅游线路和大湘西精品旅游线路，支持旅游风情小镇、“美丽乡村”和国家旅游扶贫村建设，努力形成全域、全年、全业的“全产业链”的现代旅游业。侯卫东官场笔记五是建设健康产业基地。支持邵东廉桥健康养老示范乡镇和中药产业园建设，支持隆回大健康科技产业园建设，支持大祥檀香园、双清莲荷村、北塔茶元头建设高标准养老健康产业园，努力建设湘中湘西南健康养老产业基地。六是建设现代物流基地。着眼“智慧城市”建设，加快发展智慧物流、农村“互联网＋电商”和寄递物流业，加快湘西南农产品物流中心、金罗湾国际商贸城、烟草物流中心、邵东星沙物流园、武冈商都物流园、隆回城东综合大市场和农产品物流园等大型物流项目建设，积极申报全国物流标准化试点城市，着力打造湘桂黔边陲物流中心。结合保税仓、监管仓建设，启动邵阳经开区大型物流中心招商工作，建设集运输、仓储、配送、信息、交易、结算和产品展示、服务外包于一体的现代综合物流服务基地。

攻坚克难抓项目。安排市重点项目300个左右，年度投资600亿元左右，其中产业项目不少180个，年度投资不少于300亿元。怀邵衡铁路竣工通车。争取呼南高铁邵永段、呼北高速隆回至武冈段尽快开工，做好溆浦南、新化南、邵阳北三个高铁出口T字型连接快线项目前期，推进兴永郴赣铁路、市域轨道交通前期工作，加快推进邵阳站综合交通枢纽建设。争取武冈机场新增1—3条航线，开工建设武冈、新宁崀山通用航空机场，积极开展城步、邵阳县通用航空机场前期工作，努力争取邵东军民两用机场尽快获批。大力实施城网建设三年行动计划，建成110KV六岭变电站；完善中心城区35千伏及以上主干电网，推进智能电网建设；支持雅中—南昌±800千伏特高压电力线路开工建设。启动市中心城区第二水源项目建设。启动“气化邵阳”二期工程，开工建设邵阳县—隆回—洞口—武冈—新宁支线。加快全市新能源汽车充电站场运营建设。强力推进PPP项目，在“两供两治”、智能城市、健康养老等领域率先突破。用攻坚克难的决心、意志、勇气、担当和能力狠抓项目建设，不推诿、不扯皮、不拖拉，不惧困难，不怕担责，推进工作落实、项目见效。

紧盯不放抓园区。邵阳经开区瞄准千亿产业、国家级园区目标冲刺。双清区协同经开区完善政策，全力解决托管后续重大问题。大祥区着力打造国家现代农业产业园。北塔区着力打造省级服务业示范集聚区。邵东以隆源中小企业创业中心、金华湘包装印刷产业园等特色园区为基地，以智能制造技术研究院、锐科机器人技术公司为研发平台，对园区传统产业进行“智能化”改造，加快推进产业升级和品质提升。新邵循环经济产业园、新宁裘革产业园，形成主导产业集聚规模。隆回整合金银花、辣椒等优势资源，推进大健康产业园建设。洞口以辣妹子食品为龙头，加快农产品产业园建设，促成台湾新金宝电子进驻投产。武冈做强卤制品品牌，加快推进温氏、大北农、帝立德等重大项目投产。邵阳县加快学海文化产业园和油茶产业示范园建设，打造“中国茶油之都”。城步加快建设以乳制品、青钱柳茶为主导的生态产业园。绥宁着力打造以贵太太、绿洲惠康为主体的食品和新材料产业园。园区建设重在招商引资。要用好用活“省十条”、“市十条”等优惠政策。支持鼓励异地邵阳商会建设，实施“邵商总部、产业、资本、市场、人才、科技、旅游、社会公益”八大回归行动。围绕“产业链”抓招商，以龙头企业为核心，引进上下游强链延链补链企业，培植产业集群。重点瞄准“三个500强”，引进亿元以上项目150个；各县市区力争每年至少引进1家500强先进制造企业进驻或设立分支机构。

各位代表，基础薄弱、发展落后是我市最大的市情。悠悠万事，发展最重。我们唯有锐意进取、埋头苦干，把产业做大、把实力做强，才能确保全省第二方阵“领头雁”的地位，最终实现跻身全省第一方阵、重振邵阳雄风的宏伟目标！

（二）凝心聚力，深化改革创新开放

全面深化改革。以中央、省委、市委深化改革精神为指导，统筹推进各项改革工作。推进供给侧结构性改革。落实“三去一降一补”措施。去产能，坚决关闭高污染、高能耗、低附加值企业。去库存，加强房地产用地调控和市场监管，促进房地产回归居住功能；市政项目建设和棚改，货币化安置力争达到80%。去杠杆，降低企业负债率，推进企业债务重组，支持银行、资产管理机构和企业开展市场化债转股；加大股权融资力度，盘活企业存量资产和闲置资产，加快处置“僵尸企业”。降成本，切实降低企业融资、用工、用能、物流、制度性交易、清算退出等成本，加快推进“三供一业”分离移交。补短板，把着力点放在脱贫攻坚、培育实体经济、壮大县域经济、加强环境治理、生态修复、创新开放、转型升级和公共服务上。推进财税金融改革。深化预算管理制度改革，强化政府预算全口径大统筹。启动“新财源建设工程”，严格控制并逐步降低非税收入占比；取消非税收入收支挂钩，增强政府可用财力。加大地方金融和政府债务监管，整合政府融资平台公司，开展县乡政府性债务清理核查。落实新型金融业态监管措施，抓好非法集资、民间融资、各类交易场所、互联网金融等专项整治，坚决守住不发生区域性和系统性金融风险和债务风险的底线。推进“放管服”改革。继续简政放权，出台行政审批事中事后监管制度，推行市场准入负面清单制度。推进事业单位改革，加快中介机构和行业协会商会脱钩。推动商事制度改革，“多证合一、一照一码”更加协调同步。深化以负面清单为核心的外商投资管理制度改革，在市场体系建设中建立公平竞争审查制度。推进农业农村改革。以农村土地“三权分置”为核心，推进农村集体产权制度改革，加快农村土地依法依规流转，培育新型农业经营主体和服务主体。推动集体经营性土地入市和宅基地制度改革，深化户籍制度改革，加快农业转移人口市民化。

突出创新引领。强化企业自主创新。大力培育高新技术企业、科技型中小企业，新增高新技术企业20家以上，R＆D经费支出占GDP比重1.4%以上，高新技术产业增加值占GDP比重18%以上。实施质量强市和品牌强市战略，支持企业开发新产品、新技术、新工艺、新服务，引导企业发扬“工匠精神”，全面提升产品质量；支持优质企业创名牌、树品牌，打造“百年老店”、著名商标、地理标志产品。加强创新平台建设。依托科技经济专家顾问团，深化与国家科研院所、高校等研发机构合作，组织实施一批产学研协同创新项目，支持组建一批工程技术研究中心和企业研发中心，培育发展一批科技企业“孵化器”和区域性“众创空间”，打造“技术链＋产业链＋市场链”集成创新平台体系。激发创新创造活力。落实和完善“双创”政策，构建经开区、产业园、大学科技园、大学生创业基地、创客空间等多方协同的创业创新机制。实施人才强市战略，致力引进一批科技领军人才和高水平创新团队，大力培养一批企业家人才队伍和高技能产业工人队伍，建设一支结构合理、素质优良的创业创新人才队伍。

加快开放崛起。立足“一带一部”新定位，积极推进对接500强提升产业链、对接“新丝路”推动走出去、对接自贸区提升大平台、对接湘商会建设新家乡、对接“北上广”优化大环境“五大开放行动”，大力发展开放型经济。着力推动“邵品出境”和邵阳—东盟文化旅游联盟。积极引导打火机、农产品、小五金、箱包、皮草、裘革等产业申报国家级和省级外贸转型升级出口示范基地，推动洞口、新宁建设省级出口食品、农产品安全示范区。实施外贸“破零倍增”工程，加快外贸综合服务体建设，推进园区保税仓改革运营试点，建设一批保税仓、出口监管仓和进口商品交易展示店，完成市进出口通关服务中心主体工程，加快邵阳公路口岸建设，支持企业开拓国际市场。大力引进各类外贸和跨境电商企业在邵设立跨境电商展示交易中心，加快发展“互联网+商贸流通”。鼓励支持优势产业、优质企业组建联盟“抱团出海”和“借船出海”，加强与央企、省属企业和境外湖南园区的联系对接，发挥龙头企业和行业协会的引领作用，带动我市产品、技术和劳务输出。

各位代表，今年是我国改革开放40周年。实践证明，改革开放是决定发展的关键一招。在新时代中国特色社会主义建设的伟大征程中，我们唯有解放思想，敢为人先，向改革要动力，向开放要红利，向创新要活力，才能在新一轮发展竞争中抢占先机，赢得主动！

（三）强基固本，推进城乡融合发展

抓好城市扩容提质。以“城市双修”为抓手，补齐城市短板，提升城市品质。科学谋划城市发展扩容。进一步优化城市规划，稳妥推进市区行政区划调整，支持邵东撤县设市。加快构建“三环”、“四线”交通网络骨架。全面启动三环线地下综合管廊和邵西大道、新城大道、金鸡路等道路桥梁建设，力争2020年全部建成通车。加快东城快线建设，启动西城快线（市区至隆回快速线）项目前期，以快速通道网络拉开“双百”城市大框架，释放主城区发展的压力。全力推动城市建设提质。坚持基础设施先行。搞好城市路网建设，实现邵水西路茅坪段、邵水东路南段、宝庆西路延伸段（提质改造）、魏源西路、邵西路中段、北塔路、中山路（扩改）建成通车，加快桃花桥、资江北路、白马大道、蔡锷大道（邵九公路）、建设北路建设，做好城市精品工程和亮点工程。实施市中心城区旅游景点建设三年行动计划，完成城区“老八景”修复，推进资江、邵水两岸保护开发。以邵阳经开区为龙头，辐射带动各类产业和园区建设，吸引更多的人才、技术、资金、信息等要素向新城区集聚，增强新城区的张力。有序推进中心城区改造。完善新老城区连接路网、电网、管网和微循环路网、电网、管网建设，打通新华南路二期、迎春路、雪峰南路延伸段等“断头路”。综合治理占道停车，增加天桥、地下通道等人车分流设施，畅通城市“肠梗阻”。将老城区设施改造和产业置换结合起来，实施“退二进三”、“退二还绿”，有序推进第二产业外迁，腾出部分土地作为城市公共绿地，形成对高端服务业的内聚效应，让老城区焕发新的活力。

实施乡村振兴战略。以“农村双改”为抓手，加快推进农业农村现代化，让农业成为有奔头的产业，让农民成为有吸引力的职业，让农村成为安居乐业的美丽家园。大力发展农村产业。协同推进三个“百千万”工程，重点扶持一批省级以上龙头企业。优化农业种养结构，做大做强生猪、柑橘、油茶、乳业、中药材等优势农产品基地，打造农产品公共品牌、产地品牌和企业品牌，增强农产品有效供给能力。以农产品精深加工业为重点，推进农村一二三产业融合发展，培育壮大休闲农业、森林康养、农村电商等新兴业态。努力补齐农村短板。加快农村水利、电网、公路、饮水等公共服务设施建设，消灭无网络村，实现宽带全覆盖。以“厕所革命”为切入点，全面整治农村村容村貌。完成省里下达的农村危旧房改造任务。实施农村公路“窄改宽”800公里，加速推进25户100人以上自然村通水泥路建设。提质改造城郊公路86.8公里，实施安保工程885公里，改造危桥56座，建成农村客运招呼站110个。全面完成1617处农村饮水工程建设，解决全市所有农村人口安全饮水问题。着力培养农村人才。鼓励更多城市优秀人才下乡创业和回家创业，支持农民工、大学生当地就业创业。加强农村干部、农民和新型主体培训，培养造就一支懂农业、爱农村、亲农民的“三农”工作队伍。加快城乡互联互通。抓好城乡客运一体化试点建设，推进各县市城乡客运一体化发展，实现县城组团、重点乡镇村的客运网络全覆盖，畅通城乡人流物流。进一步做好农网提质改造。推进用水城乡联网同价，用气同步规划、同步实施。推动医疗、教育、养老、社会保障、文化体育、就业培训等社会事业向农村延伸，全面开启农村生活城镇化、城乡保障一体化、区域产业现代化的新道路。

各位代表，发展不平衡、不充分是我市经济社会的突出矛盾，集中表现为城乡发展不平衡和农村发展不充分。我们唯有加快补齐农村这块短板，帮助农民实现像城里人一样生活的千年梦想，才能让广大群众切身感受到党和政府的阳光雨露和新时代中国特色社会主义的优越性。这是贯彻以人民为中心的发展思想的根本要求。

（四）齐抓共管，努力建设美丽邵阳

创建全国文明城市。坚持创建为民、创建惠民。抓“硬件”、强“筋骨”，在巩固“创国卫”成果基础上，进一步夯实创建基础，大力开展占道经营、违章建筑、环境脏乱差、空气质量差和社会治安秩序、城市交通秩序、文化市场及校园环境、乱采乱挖乱排等重点整治，全面提升城市品质。抓“软件”、聚“灵气”，推进群众性精神文明创建，深化文明社区、文明村镇、文明单位、文明家庭创建活动，加强社会主义核心价值观和社会公德、职业道德、家庭美德、个人品德教育。建设社会诚信体系，建立公共信用共享平台，强化守信联合激励和失信联合惩戒机制，推动形成重信、守信的社会风尚，为经济社会持续健康发展输送大“资本”、正能量。

创建国家森林城市。国家林业局已批复同意我市创建国家森林城市。以此为起点，优化森林城市布局，同步推进生态建设和生态保护，切实抓好绿色通道、绿色水道、美丽乡村、生态文化等九大生态工程建设。持续推进城市中心园林化、城郊地区森林化、江河道路林网化、社区庭院花园化、产业经济生态化。坚持造林与造景、造园、造福结合，绿化与美化、彩化、香化结合，开展道路增绿、景区增绿、社区增绿和系统增绿。以“四边五年”绿色行动和城区绿化攻坚为抓手，以宝庆森林公园整体开发为支撑，力争三年内建成国家森林城市。

创建国家健康城市。发挥“五馆一中心”和体育中心等平台作用，打造健康主题公园，发展健康文化。推进健康单位、健康社区、健康家庭等健康细胞建设，构建健康社会。加强传染病、地方病、职业病等监测和预防控制，提升重大疾病和突发公共卫生事件预警和处置能力，优化健康服务。开展无烟单位创建活动，倡导绿色低碳出行，组织形式多样的全民健身活动；依托公园、景点等休闲场所，建设健康广场、健康步道，打造城市社区15分钟健身圈，培育健康人群。同步推进国家健康城市、全国文明城市、国家森林城市创建，带动县（市）乡（镇）创卫、创文，营造文明卫生健康环境。

做美南山国家公园。突出抓好公园总体规划编制、自然资源确权登记、生态补偿、公园立法以及生态保护性基础设施建设，修复“碎片化”，保护“原真性”。用好36亿第一期国家项目资金，重点做好草山植被生态恢复、核心保护区生态移民搬迁和水电风电等涉矿涉企产业逐步有序退出，加强公园生态体验、科普教育以及绿色发展相配套的基础设施建设，有效保护和合理利用现有资源，建立科普教育与生态旅游协调、资源保护与区域发展统一的国家公园建设新模式，不断提升影响力和知名度，打造绿色邵阳新名片。西部几县要利用南山国家公园品牌，依托自身优势，做好生态旅游大文章。

打好污染防治攻坚战。认真落实“党政同责、一岗双责、失职追责”规定，开展领导干部自然资源资产管理和生态环境保护离任审计。严格执行环境影响评价和环保“三同时”制度，坚决杜绝新上污染项目。加大对去年中央、省环保督察交办问题整改落实的跟踪检查，确保件件真整改、事事真落实，不反弹、不死灰复燃。开展大气污染防治专项行动，完成国电宝庆煤电有限公司1#机组超低排放改造，继续推进市城区煤改气工程；加大机动车尾气污染物检测，再投放新能源公交车400台；加强对建设工地扬尘、道路扬尘、露天堆场及货物扬尘的综合治理，控制工业粉尘污染，全面禁止秸秆焚烧，将市城区禁放烟花爆竹向各城镇延伸，扫除十面“霾”伏，坚决打赢蓝天保卫战。全面落实“河长制”，实行“河”“岸”共治，加大资江、邵水、红旗河水污染治理，强化全流域生态环境保护。实行城市建成区生活污水全截污、全处理、全达标，黑臭水体治理全覆盖。推进龙须塘片区综合治理，推进“退城入园”，加快区域内排污管网配套建设和“两沟”清淤疏浚、“两路”升级改造。推进城乡垃圾转运站、垃圾无害化处理，搞好垃圾分类处理和餐厨垃圾资源化利用。建立市县乡村四级环境监管网格化体系，严查严处各类干扰监测的违法行为。启动市级环保督察，对重点地区、重点行业开展机动式、点穴式专项督察，让污染企业和污染行为无处遁形。

各位代表，绿水青山就是金山银山。生态建设是最大的民生福祉，生态资源是最宝贵的公共资源。我们唯有牢牢守住环境底线，像保护眼睛一样保护生态，像善待生命一样善待环境，让邵阳大地青山常在、碧水常流、空气常新，我们的发展才有意义，才不辜负生育、养育我们的净土乐园、父老乡亲！

（五）精准扶贫，切实保障改善民生

决战决胜脱贫攻坚。紧扣“一高于一接近”、“两不愁三保障”目标和“六个精准”要求，用绣花功夫推进脱贫攻坚，确保绥宁、武冈、新邵3个县市脱贫摘帽、400个贫困村出列、24万贫困人口脱贫。全面落实“三走访、三签字”脱贫责任。改善基础设施和公共服务，推动特色产业、劳务输出、生态保护、教育和健康扶贫。实施贫困村整体提升工程，增强贫困地区和贫困群众自我发展能力。开展涉农资金整合，引导金融和社会资本投入，鼓励龙头企业、合作社与贫困户建立稳定利益联结机制，发展壮大乡村旅游、光伏发电等扶贫产业。大力推进电商扶贫，力争所有贫困村都建有村级服务站和电商扶贫示范网店。易地扶贫搬迁34473人，让贫困户搬得出、稳得住、逐步能致富。着力抓好驻村帮扶，落实单位、人员责任。推进专项扶贫、行业扶贫，动员更多社会组织和爱心人士参与社会扶贫。不让一个孩子因贫辍学，不让一个困难群众因病返贫，让所有贫困群众早日踏上小康路、过上好日子，做到脱真贫、真脱贫。

抓紧抓好民生实事。扎实做好稳岗就业工作，支持农民工返乡创业。积极开展职业技能培训和创业培训，做好高校毕业生、就业困难人员、农村转移劳动力、退役军人等重点人员的就业工作。加大社保体系建设，落实城乡养老保险制度衔接，完善城乡居民基本医疗保险制度整合。扩大社会保险覆盖范围，建立疾病应急救助基金。坚持“四个最严”要求，全力抓好食品药品安全，实现食美药安。不断扩大住房公积金制度覆盖面，逐步推进住房公积金向农民工、个体工商户和外来常住人口延伸。继续实施保障性住房安居工程，完成各类棚户区改造30506套。积极应对人口老龄化，构建养老、孝老、敬老政策体系和社会环境，推进医养结合，加快老龄事业和产业发展。

优先优办社会事业。坚持教育先行。统筹推进城乡义务教育一体化发展，促进各级各类教育均衡发展。推进职教新城建设。加快市一中搬迁建设，确保今年秋季开学投入使用。支持鼓励社会力量办学，落实房地产开发企业履约建校责任，加快解决城区“大班额”问题。实行“医保、医药、医疗联动”，做好医疗服务保障。坚持计划生育基本国策，提升妇幼保健、计划生育服务能力。实施文化惠民工程，着力提高公共文化服务水平。开展全民健身活动，促进群众体育与竞技体育协同发展。加快市体育中心项目建设，当好东道主，高质量、高水准办好湖南省第十三届运动会。鼓励社会资本进入教育、医疗、健康、文化等领域，加快形成统一开放、公平竞争的市场体系。认真贯彻落实强军目标，扎实推进双拥、国防动员、兵员征集和后备力量建设工作创新发展。支持各民主党派、工商联、无党派人士及各人民团体工作。

各位代表，人民群众对美好生活的向往就是我们的奋斗目标。我们唯有用心用情用力谋民生之利，解民生之忧，在幼有所育、学有所教、劳有所得、病有所医、老有所养、住有所居、弱有所扶、贫有所帮上下功夫，才能保障全市人民在共享发展中有更多获得感，才无愧于830万邵阳人民的信任和重托！

（六）履职尽责，积极维护安全稳定

创新社会治理。建立健全党委领导、政府主导、社会协同、公众参与、法治保障的社会治理体制，形成政府治理和社会调节、居民自治良性互动，确保综治民调进位不退位。深入开展“七五”普法活动，扎实做好人民调解和社区矫正工作。全面开展“信访法治年”专项活动，创新开展“三无县市区、乡镇村（社区）”创建活动；推行“以村为主”工作模式，设立“一村一接访中心”（原群众工作站）、“一村一信访员”、“一村一信访调解委员会”，最大限度把矛盾化解在初访、解决在基层。加快村级综合服务监管平台建设，探索建立村级辅警、河段长、护林员、保洁员等聘用人员联合出勤机制，进一步提升乡村基层组织社会综合治理能力，推动形成良好社会秩序。

强化安全生产。坚决落实安全生产党政领导责任、部门监管责任和企业主体责任，健全重大隐患治理“一单四制”动态化管理，进一步强化安全管理、防范、监督和检查，坚决杜绝重大及以上事故，确保安全生产形势持续稳定。推进重点领域、重点行业安全生产智能化监测。运用互联网等新技术，构建生产、运输、存储、销售、使用等全过程、无缝隙监管体系，实现对各类安全生产风险自动识别、预警、预防，有效减少生产安全事故发生。

维护社会稳定。健全社会稳定风险评估机制和应急管理机制，提高突发公共事件防范处置能力。发挥“一村一辅警”作用，完善社会矛盾排查预警和多元化解机制。依法做好民族宗教事务管理工作，深入开展民族团结进步创建“六进”活动。加强社会组织管理，建立社会组织“异常名录”和“黑名单”。加强社会治安防控体系建设，继续开展打黑除恶专项斗争，严厉打击各类暴恐犯罪、刑事犯罪、金融犯罪、网络犯罪以及黄赌毒等一切违法犯罪活动，不断提升社会公众的安全感。

各位代表，发展的重任、人民的期盼，对政府自身建设提出了更高的要求。我们将始终本着对人民、对事业高度负责的态度，努力打造为民务实清廉的政府。强化为民宗旨。认真开展“不忘初心、牢记使命”主题教育，把执政为民贯穿政府工作始终。加快政府职能转变，创新公共服务供给方式，完善政府购买服务清单，集中更多财力用于公共服务和民生改善。坚持放权于企、让利于民，正确处理政府与市场的关系，不断优化“互联网+政务”和“互联网+监管”，让群众和企业少跑腿、好办事。严格依法行政。坚持领导干部带头尊法学法守法用法，坚持运用法治思维和法治方式推动工作，坚持法定职责必须为、法无授权不可为，认真执行重大行政决策程序规定，让权力在阳光下运行，确保决策制度科学、程序正当、过程公开、责任明确。依法接受人大及其常委会的监督，自觉接受人民政协的民主监督，认真做好人大议案、代表建议和政协提案办理工作。主动接受司法机关监督，接受社会公众监督和新闻舆论监督，充分听取民主党派、工商联、无党派人士和各人民团体的意见。切实改进作风。坚持求真务实、真抓实干，不搞虚假政绩、不搞数字攀比、不搞形象工程。把雷厉风行和久久为功有机结合，一件接着一件办，一年接着一年干，办一件，成一件。健全问责处罚机制，力戒敷衍了事、推诿扯皮、为官不为，坚决整治庸政、懒政、怠政。恪守廉洁从政。认真执行党风廉政建设责任制，切实履行主体责任。认真落实“一岗双责”，深化廉政风险防控，坚持不懈纠正“四风”。坚持用制度管权、管事、管人，强化行政执法和审计监督，严管公共资金、国有资产资本和公共资源交易。坚决反对腐败，坚持无禁区、全覆盖、零容忍，严查重点领域和关键岗位腐败，严惩侵害群众利益的不正之风和害群之马，严治“雁过拔毛”的微腐败、尤其是涉及扶贫领域的违法违规和腐败行为。

各位代表，实干成就梦想，奋斗铸造辉煌。让我们紧密团结在以习近平同志为核心的党中央周围，在中共邵阳市委的坚强领导下，紧紧依靠和团结全市人民，凝心聚力、奋发有为，不断开创经济社会发展新局面，为建设富饶美丽幸福新邵阳而努力奋斗！
